# Bioelectrical Impedance Analysis (BIA) detects body resistance increase in dogs undergoing blood donation

**DOI:** 10.1007/s11259-024-10555-1

**Published:** 2024-09-27

**Authors:** Noemi Nisini, Andrea Corda, Francesco Birettoni, Arianna Miglio, Maria Teresa Antognoni

**Affiliations:** 1 Department of Veterinary Medicine, University of Perugia, Perugia, Italy; 2 Department of Veterinary Medicine, University of Sassari, Sassari, Italy

**Keywords:** Bioelectrical impedance analysis, Dog, Phase angle, Blood donation, Total body water

## Abstract

Using raw parameters of bioelectrical impedance analysis (BIA) and derived Phase Angle (PhA) has emerged as an alternative to conventional predictive BIA for body fluid volumes and body composition estimation in human patients, offering an approach independent of predictive equations. Classic predictive equation-based BIA remains unsuitable for veterinary clinical practice due to the high morphological variability within the canine species. This study aimed to validate the feasibility of BIA in dogs and assess its utility in detecting changes in body fluid volumes, particularly in blood donation. Sixty healthy blood donor dogs and ten controls were longitudinally assessed using BIA raw parameters with a portable analyzer at a 50 kHz frequency. Measurements were performed before and after blood donation in donor dogs, whereas in controls, they were carried out at two time points, approximating the timing of a donation. Significant increases in raw bioelectrical parameters were observed after blood donation in the donor group, while differences were not statistically significant in controls. Hematocrit values did not significantly differ between the two time points in both groups. These findings suggest the feasibility of using BIA to detect changes in body fluid volumes in dogs undergoing blood donation, highlighting its potential application in veterinary medicine. Further research should explore the applicability of BIA in spontaneous pathological conditions involving fluid loss or retention and investigate the potential utility of PhA as a prognostic or monitoring tool in veterinary clinical practice. This study provides valuable insights into using BIA in canine medicine, paving the way for enhanced diagnostic and monitoring strategies in clinical practice.

**Supplementary Information:**

The online version contains supplementary material available at 10.1007/s11259-024-10555-1.

## Introduction

Maintaining a constant volume and stable composition of body fluids is essential for homeostasis. Many diseases and conditions affect body fluid balance, and disorders of body water content are common complaints in clinical practice (Asogwa and Lai 2017). Estimating total body water (TBW) content is crucial in the prognostic assessment of critically ill patients and should be considered a vital parameter (Di Somma et al. 2012). The measurement of TBW is also useful for evaluating the efficacy of therapeutic and pharmacological interventions (Shiba et al. 2020).

Both in human and veterinary clinical settings, signs and symptoms of hydration imbalances are roughly estimated using physical examination. Laboratory tests (i.e., hematocrit and total blood proteins) and imaging techniques (i.e., ultrasound and Computed Tomography) are more sensitive in indirectly detecting body fluid changes. Still, they need expensive equipment and specialized operators. They are often unsuitable for quantifying acute shifts in body fluids in critical ills and situations of rapid alteration of body fluid balance. Therefore, accurate quantification of these changes remains a challenge.

Bioelectrical Impedance Analysis (BIA) is a non-invasive, inexpensive, portable, real-time and easy-to-use diagnostic tool routinely used in human clinical practice to determine body composition and assess fluid status (Khalil et al. 2014; Moonen and Van Zanten 2021). Fluid status assessment and detection of fluid disorders with BIA is based on the theory that fluid overload will increase the conductivity of an electrical current passed through the body. In contrast, the loss of fluid will reduce this conductivity. The everyday basis of all BIA analysis systems and devices is the measurement of body impedance or bioimpedance. The impedance (Z) is the opposition to the flow of an alternating electric current passing through tissues. The Z is geometrically composed of two parameters: resistance (R, Ohm) and reactance (Xc, Ohm). The bioelectrical R represents the opposition offered by the body to the flow of the alternating electrical current and is inversely related to the water and electrolyte content, while the bioelectrical Xc is associated with the capacitive properties of the cell membrane and variations that can occur depending on its integrity (Thomasset 1962; Campa et al. 2023). Conceptually, R is inversely proportional to the amount of TBW, and Xc is more closely related to the structure and function of cell membranes.

Additionally, the capacitance causes the administered current to lag behind the voltage, and it creates a phase shift that is represented by the bioelectrical Phase Angle (PhA) (Kushner 1992). PhA obtained with BIA is an indirect parameter that reflects the relationship between body R and Xc and is calculated as the arc-tangent [Xc/R] × [180°/π] (Lukaski et al. 2019). Therefore, the PhA depends both on the capacitive behavior of the tissues, which is associated with tissue cellularity and cells size, and their pure resistive behavior, which is primarily related to tissue hydration and membrane permeability (Baumgartner et al. 1988; Selberg and Selberg 2002).

In this classic predictive BIA method, bioelectrical data obtained at one or more current frequencies and morphometric measurements are used to estimate various body compartments using predictive equations previously derived from reference data in the human population.

The use of BIA raw parameters or derivatives of raw data, such as the PhA, gave rise to detailed data analyses and extended the application in acute body fluid imbalances detection in human critical care setting (Piccoli 2010; Malbrain et al. 2014) since they are independent of conventional regression equations for estimating body composition and fluid status. The usefulness of BIA raw parameters, particularly the PhA, and their prognostic role in the longitudinal assessment of patients with body fluid imbalances has thus gained attention (Piccoli et al. 1994).

Yaguiyan‐Colliard et al. (2015a) proposed BIA-derived regression equations to estimate TBW in dogs. Still, these failed to be accurate in dog breeds different from Beagle in a subsequent study conducted by the same research team (Yaguiyan-Colliard et al. 2015b) due to the extreme variability among dog breeds in morphology and body size. These extreme morphological differences affect the passage of the electrical current through the body and have severely affected the possibility of developing and validating predictive equations for estimating body composition and body fluid volumes with BIA.

To overcome the limitation mentioned above of the classic BIA approach in dogs, we designed a longitudinal study to evaluate the potential of BIA to indirectly track TBW variations in dogs without using regression equations but tracing the variations of raw bioelectrical parameters of Impedance (Z), Resistance (R), Reactance (Xc) and the derived Phase Angle (PhA). For this purpose, we used blood donors as an experimental canine model of acute intravascular fluid loss, performing BIA measurements in donor dogs before and after a whole blood donation. Blood donation indeed acutely perturbs body fluid homeostasis and TBW due to the controlled leak of fluid from the intravascular space, allowing a blood collection of up to 13% of total blood volume (10 ml/kg) without any clinical signs (Ford and Mazzaferro 2006; Gibson and Abrams-Ogg 2012; Ferreira et al. 2015).

This study aimed to validate the feasibility of BIA in dogs and investigate if BIA raw parameters could be used to detect and track changes in body fluid volumes in dogs undergoing blood donation.

## Materials and methods

### Dogs

Sixty healthy blood donor dogs voluntarily enrolled in the blood donor program of the Veterinary Teaching Hospital of Perugia from April 2021 and June 2022 were included. No specific breed requirements were considered for inclusion in the study, and as a primary criterion, we used the minimum weight (16 kg) necessary for eligibility in the donor program. Nonetheless, dogs with skin lesions or abnormalities in electrode attachment points were further excluded. Dogs were considered healthy based on history, physical examination, cell blood count, and chemistry panel results.

In addition, ten healthy dogs from Hospital staff were enrolled as control subjects. In this group, we performed all the study protocols under the same conditions as the donor group, except that the dogs did not donate blood.

All experimental protocols in this study adhered to the European Union guidelines and were approved by the University of Perugia Bioethical Committee (protocol n°67668, 13/04/2021).

### Bioelectrical impedance analysis

**Fig. 1:**
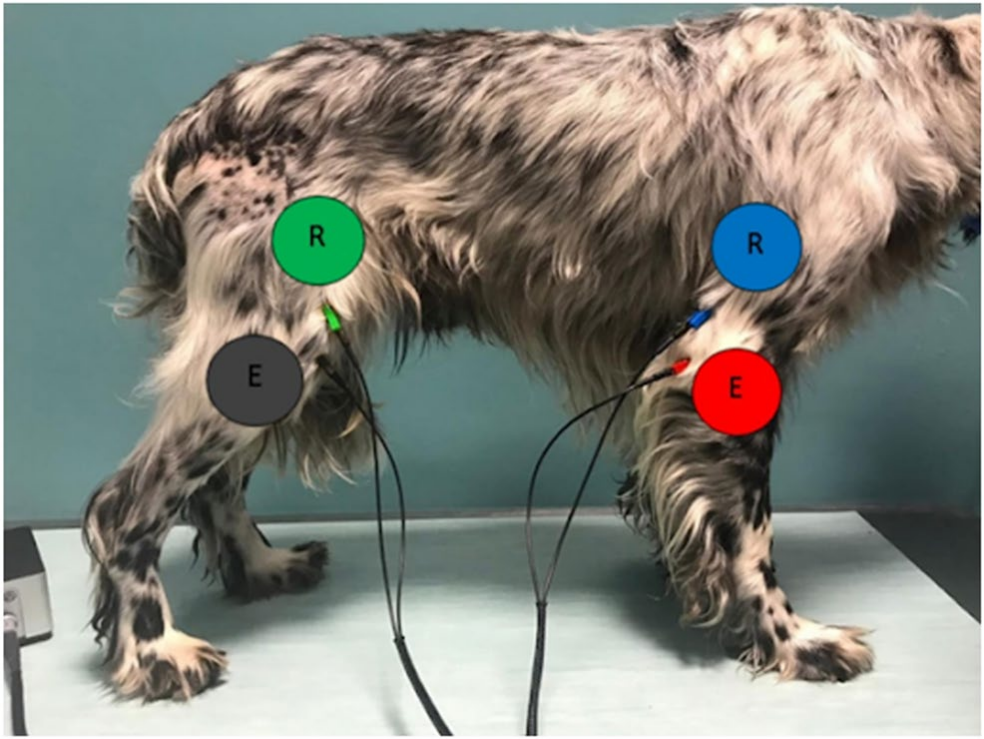
Whole-body right tetrapolar electrode configuration for BIA analysis. Two emitting current-injecting electrodes (red and black) are placed dorsal to the elbow and dorsal to the patella. Two receiving voltage-sensing electrodes (blue and green) are applied 3.5 cm dorsally to their emitting electrodes. An imperceptible alternating sinusoidal electric current of 800 μA at an operating frequency of 50 kHz was used through the current-injecting distal pair

Food was restricted 12 h before the blood donation and BIA measurement session. Water was restricted one hour before BIA and during all the study protocols. All BIA protocol was carried out in non-sedated dogs, gently restrained in a natural standing position on a non-conductive insulating mat. To avoid electric interferences during measurements, care was taken to ensure that the dog could not contact any electrically conductive object. For this purpose, any collar or harness was removed, and dogs were handled using latex gloves, ensuring all four feet touched the ground. We obtained three consecutive BIA measurements immediately before donation, using a portable, wireless, phase-sensitive impedance analyzer (Biosmart® EX.516(XX), Eupraxia, Italy) at a single frequency of 50 kHz. We adopted a right whole-body tetrapolar electrode configuration previously validated in dogs (Yaguiyan-Colliard et al. 2015a, b). In this configuration one pair of current-injecting electrodes were applied by crocodile clips on the skin dorsal to the right elbow and dorsal to the patella of the right hindlimb. one pair of current-injecting electrodes were applied by crocodile clips on the skin dorsal to the right elbow and dorsal to the patella of the right hindlimb. The two voltage-sensing electrodes were positioned 3.5 cm dorsally to their respective current electrodes (Fig. 1).

Electroconductive gel was applied to the skin at the electrode attachment points. The hair was not clipped but accurately moved aside to expose the dog’s skin. The alternating current was applied using the distal pair of electrodes, and the proximal pair detected the voltage drop. BIA raw parameters obtained from this set of electrodes are referred to as “whole-body”; they were directly sent from the analyzer to the analysis software.

Immediately after the donation, we performed another set of three consecutive whole-body measurements. Each measurement lasted approximately 5 s and allowed to obtain in real-time whole-body BIA raw parameters of Z (Ohm), Xc (Ohm), R (Ohm), and PhA (°), which was directly calculated from the analyzer. Unless Biosmart ® is a multifrequency device and can operate at two different frequencies (50 and 100 kHz), we considered only the mean values of measurements recorded at 50 kHz before and after donation for the analysis. We chose this analysis frequency according to the existing dog literature and because, according to human data, 50 kHz is the frequency both R and maximum Xc are best measured, and bioelectrical parameters measured at 50 kHz are most frequently used (Moonen and Van Zanten 2021; De Borba et al. 2022; Bellido et al. 2023).

**Fig. 2:**
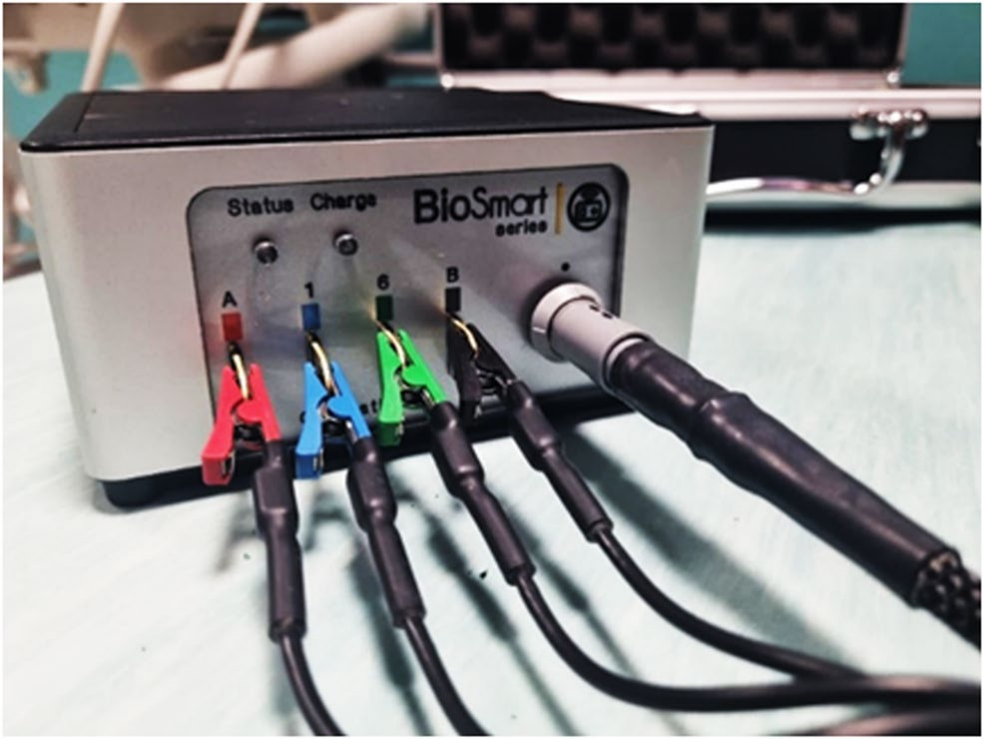
Biosmart ® EX.516(XX) calibration test

Healthy control dogs were studied in the same manner. In this group we performed 3 sequential BIA measurements at T0 and T1 (approximately 20 min after T0). 50 kHz bioelectrical parameters were directly sent from Biosmart ® to the dedicated software, averaged, and used for the analysis. A calibration test was performed before each measuring session in both groups using the supplied test circuit, which ensures system and data accuracy (Fig. 2). For both donor and control groups, the 3 sequential whole-body BIA measurements recorded at 50 kHz before and after donation and at T0 and T1, respectively, were averaged and used for the analysis.

### Blood testing

Blood samples (2 mL) were collected in EDTA tubes from the cephalic vein for cell blood count in both groups. The donors' collection was performed immediately before and after donation; in the control group, two samples were taken 20 min apart, approximating the donation timing. Hematocrit (HCT) percentages before and after donation for donors and at T0 and T1 for controls were then assessed (hematology analyzer, Sysmex XT-2000iV; Sysmex, Kobe, Japan) and used for the analysis.

### Morphometric analysis

Each dog was weighed before blood donation, and its body weight (BW) was recorded. According to previous studies (Yaguiyan-Colliard et al. 2015a, b), with the dog in standing position, the body length (BL) from the external occipital protuberance to the base of the tail was measured with a flexible tape measure; rib cage circumference (RC) was measured at the level of the xiphoid process and the circumference of the abdomen (AC) at the level of the umbilicus with the dog at full expiration. BW was measured to the nearest 0.1 kg, and all other measurements were measured to the nearest 0.1 cm.

### Statistical analysis

Qualitative variables were described by absolute and relative frequency. Quantitative variables were summarized by mean and standard deviation (SD) or median and interquartile range (IQR), depending on their distribution, which was evaluated using the Shapiro–Wilk test. Differences between donors and control groups were assessed using the Mann–Whitney *U* test (for quantitative variables) and the Fisher exact test (for qualitative variables). Differences in paired quantitative variables (before *vs.* after in the donor group and T0 *vs* T1 in the control group) were assessed using the Wilcoxon matched-pairs signed-rank test or the Student's t-test for paired data. P values less than 0.05 were considered statistically significant. Statistical analysis was performed using Stata 14.2 software (StataCorp, Lakeway Drive, College Station, Texas, USA).

## Results

Sixty donor dogs (33 males and 27 females) and ten control dogs (4 males and 6 females) were used. Donors’ median (IQR) age was 4 (2—6) years. Median (IQR) BW was 19.75 (17.25—23), and the mean (SD) of blood drawn was 299 (59) ml. The donor group was morphometrically homogeneous and mainly composed of medium-size breed dogs, with a prevalence of hunting breed dogs. Mean (SD) BW was 21 (5.3) kg, mean (SD) BL was 72.1 (6.7) cm, mean (SD) RC was 64.7 (6) cm, and mean (SD) AC was 50 (5.7) cm.

Among the 60 donor dogs, 16 were Ariégeois, 11 English Setters, 8 Italian Hounds, 7 Maremma Hounds, 7 Kurzhaar, 3 mixed-breed dogs, 2 Galgo, 2 Pitbull, and 1 each Italian Beagle, Petit Bleu de Gascogne, Golden Retriever, and Akita Inu.

Raw BIA parameters before donation were:

- Mean (SD) Z at 50 kHz: 201.6 (31.9) Ω
- Mean (SD) R at 50 kHz: 200.7 (31.8) Ω
- Mean (SD) Xc at 50 kHz: 19.6 (4.9) Ω
- Mean (SD) PhA at 50 kHz: 5.6 (1.2) °

Control dog breeds were 2 Italian Hounds, 2 mixed-breed dogs, 1 Ariégeois, 1 English Setter, 1 Grand Bleu de Gascogne, 1 Gascon Saintongeois, 1 German Shepherd, and 1 Corso Dog. The control group’s median (IQR) age was 5 (1–7) years, and the median (IQR) BW was 23.5 (17.5—29) kg. As reported in Table 1, differences between morphometric parameters of BW, BL, RC, and AC were not statistically significant between the two groups.

**Table 1:** Mann–Whitney *U* test (quantitative variables) and Fisher exact test (qualitative variables) were used to compare morphometric characteristics between the two study groups

	Donors group (*n* = 60)	Control group (*n* = 10)	*p*-value
Sex, males, *n* (%)	33 (55)	4 (40)	0.49
Age, years, median (IQR)	4 (2–6)	5 (1–7)	0.71
BW, Kg, median (IQR)	19.75 (17.25–23)	23.5 (17.5–29)	0.29
BL, cm, median (IQR)	71 (68–77.5)	75.5 (67–81)	0.38
RC, cm, median (IQR)	65.5 (61–69)	67 (56–70)	0.76
AC, cm, median (IQR)	50 (45–53)	50.5 (45–59)	0.57

*IQR* interquartile range, *BW* body weight, *BL* body length, *RC* rib cage circumference, *AC* abdominal

In the donor group, the median (IQR) HCT before donation [43.7 (41.5–46.5)] did not differ significantly compared to the post-donation median (IQR) value [44.2 (41.6–46.8)]. As reported in Table 2, the raw bioelectrical parameters of Z, R, Xc, and PhA, analyzed at 50 kHz in the donor group, were significantly higher after blood donation.

**Table 2:** Student's t-test for paired data results comparing donor group whole-body BIA raw parameters obtained before and after blood donation

	Donors group (*n* = 60)		*p*-value
BIA raw parameters at 50 kHz	Before donation	After donation	
Z, Ω, mean (SD)	201.6 (31.9)	208.6 (33.4)	0.02*
R, Ω, mean (SD)	200.7 (31.8)	207.4 (33.2)	0.03*
Xc, Ω, mean (SD)	19.6 (4.9)	22 (5.6)	0.00*
PhA, °, mean (SD)	5.6 (1.2)	6.1 (1.3)	0.00*

*Z* impedance, *R* resistance, *Xc* reactance, *PhA* phase angle; * statistically significant difference

In the control group, the median (IQR) HCT at T0 [45.8% (42.9–46.8)] did not differ significantly compared to the median (IQR) value at T1 [45.5% (42.4–46.8)].

As reported in Table 3, the raw bioelectrical parameters of Z, R, Xc, and PhA, analyzed at 50 kHz in the control group at T0 and T1 time points, were not significantly different.

**Table 3:** Wilcoxon matched-pairs signed-rank test results of whole-body raw parameters of the control group obtained at T0 and T1 time-points

	Control group (*n* = 10)		
BIA raw parameters at 50 kHz	T0	T1	*p*-value
Z, Ω, median (IQR)	195.4 (163.5–210.7)	179.1 (161.5–209.8)	0.07
R, Ω, median (IQR)	194.8 (162.9–209.5)	178.4 (161–208.1)	0.06
Xc, Ω, median (IQR)	17.13 (14.47–20.52)	18.9 (12.8–21.2)	0.33
PhA, °, median (IQR)	5.17 (84.72–5.86)	5.84 (4.56–6.10)	0.17

*Z* impedance, *R* resistance, *Xc* reactance, *PhA* phase angle

## Discussion

The results of the present study showed that BIA analysis in dogs is feasible and can detect changes in body fluid volumes in subjects undergoing blood donation.

We demonstrated that all raw BIA parameters (Z, R, Xc, and PhA) increase after a mild controlled blood loss in dogs. This occurred following the increase of total body impedance due to the decreased TBW content, as documented in human blood donors by Scheltinga et al. (1991). The serial longitudinal assessment of raw BIA parameters could be useful to monitor the evolution of diseases and conditions characterized by fluid imbalances and overcome the significant limitation of BIA in this field represented by the application of regression equations requiring both BIA raw data and morphometric parameters and need to be validated against invasive reference methods for body composition and fluid status assessment.

In this study, we found significantly different values for BIA raw parameters recorded before and after blood donation, while no differences were found in HCT values. HCT has a limited clinical early predictive value in the course of acute hemorrhages with an acute drop in circulating volume of < 20% of total blood volume since it is often compensated through short-term modification of the cardiovascular system leading to vasoconstriction, increased cardiac contractility, increased heart rate and neurohormonal-mediated restoration of the average vascular volume (Nelson and Swan 1972). Moreover, in dogs, the spleen acts as a reservoir for RBC since as much as 20% of the circulating RBC mass can be autotransfused after splenic contraction to the blood circulation in response to shock, hypoxia, acidosis, and acute blood loss, contributing to counterbalance HCT decrease (Nelson and Swan 1972). In an experimental study on acute loss of up to 40% of total blood volume in a group of five anesthetized Beagles (Lynch et al. 2016), authors found a mild albeit significant decrease in HCT after hemorrhage. Authors expected even a larger reduction of HCT in their study and hypothesized that splenic contraction may have counterbalanced HT decreases. We can, therefore, suppose that splenic contraction and accelerated erythrocyte release into the circulation during the blood removal in not splenectomized dogs may have affected our HCT decreases as well, limiting its clinical value in acute hemorrhages. In this study, post-donation HCT values were assessed immediately after donation. Therefore, the intravascular fluid replacement and restoration of the average vascular volume, which determines the dilution of the red blood cell mass and the reduction of the HCT value, have not yet occurred. Lastly, our donors lost less than 20% of their total blood volume during blood donation. Therefore, HCT is confirmed to be a parameter with a low predictive value in acute blood losses < 20% of total blood volume.

In the control group, the study protocol was carried out under the same conditions as the donor group except for the blood donation. Differences between bioelectrical parameters recorded at T0 and T1 were not statistically significant. We also didn’t find any statistically significant differences in HCT between T0 and T1 in this group.

Due to the high morphological variability between breeds, reference intervals for raw BIA parameters are still unavailable for dogs. This study's donor group comprised healthy adult hunting dogs with common morphometric traits. Raw whole-body BIA parameters of Z, R, Xc, and PhA recorded in our group of donor dogs before donation could provide the foundation for further and more extensive studies to build bioelectrical reference intervals for medium-sized hunting dogs. Yaguiyan-Colliard et al. (2015a) reported a mean R-value of 152.04 ± 24.32 Ω and a mean Xc of 37.26 ± 11.80 Ω for their group of healthy adult beagle dogs; this parameter was recorded with a 50 kHz single-frequency analyzer with the same electrodes configuration as our study and the dogs had a mean BL of 53.16 ± 5.11 cm. In our group of healthy adult medium-sized breed dogs with a mean BL of 72,1 ± 6,7 cm, we found a mean R of 201 ± 32 Ω and a mean Xc of 19,6 ± 4,9 Ω. Differences in our results are reasonably due to the morphological differences between our study groups, as the higher mean BL of our dogs could explain the higher value of R that we have recorded since the increase in body length affects the passage of the electrical current with a greater resulting R.

To our knowledge, this is the first paper reporting data values for PhA in dogs. In our study, parallel to the intravascular fluid loss, we observed a statistically significant increase of the PhA from 5.6° to 6.1° in donor dogs. Losses of body fluids alter the passage of an electrical current through the body, with increases in bioelectrical body resistance (R). Since the PhA is obtained directly from the BIA raw impedance data as the arctangent value of the ratio of Xc to R × 180°/π, the intravascular fluid loss of our donor dogs is reflected in the higher values of PhA after the blood donation since both R and Xc have increased. Further studies are needed to investigate whether this could be reproduced in spontaneous pathology involving fluid loss and to assess if PhA could be helpful, as in humans, in tracking decongestive treatment effectiveness in dogs with pathologic fluid retention. In our preliminary study in dogs affected by cardiogenic ascites secondary to right-sided congestive heart failure, we observed a significant increase in PhA after the resolution of the congestive status (Nisini et al. 2021).

Scientific interest in the usefulness of PhA has been increasing in recent years since it has shown to be highly predictive of impaired clinical outcomes and mortality in a variety of diseases (Norman et al. 2012; Bellido et al. 2023), and it has gained attention since it does not rely on predictive equations and offers an alternative to predictive BIA approach, which is known to be inaccurate and error-prone in diseased people with an altered body fluid status (Kyle et al. 2004; Lukaski et al. 2019; Ugras 2020).

In cardiology settings as well as in nephrology, hepatology and most recently in COVID-19 patients (Ontanilla-Clavijo et al. 2019; Cornejo-Pareja et al. 2022; Kim et al. 2022; Osuna-Padilla et al. 2022) PhA has been proposed as a possible biomarker, with lower values linked to increased morbidity and mortality and worse outcomes (Ontanilla-Clavijo et al. 2019). PhA could effectively detect fluid overload of patients with CHF during the hospitalization course (McDonagh et al. 2021), thus allowing the evaluation of their re-compensation process.

Similar results were obtained from De Ieso et al. in patients with CHF, where increased PhA was observed during the hospitalization after intensified diuretic therapy (De Ieso et al. 2021). PhA was found to be significantly lower in heart failure patients with peripheral edema and fluid retention while increased soon after therapies for restoration of clinical stability (Colín-Ramírez et al. 2006; Alves et al. 2015; Massari et al. 2016). PhA effectively showed lower values in patients in advanced New York Heart Association classes of heart failure. PhA was lower in patients in classes III-IV than in those in classes I-II (Castillo Martínez et al. 2007).

According to human data, we can hypothesize that the bioelectrical PhA could also be a valuable prognostic marker in dogs with body fluid imbalances. Further studies could establish PhA cut-off values for dogs, which could be integrated into the current monitoring protocols to identify patients at risk for developing fluid imbalances in various clinical settings and to manage therapeutic interventions and disease follow-up better.

PhA reference values and cut-offs are mandatory for assessing individual deviations from the population average. Reviewing human literature, the PhA measured at 50 kHz is most frequently used, and most reference data are available for this frequency, as this is the frequency at which both R and maximum Xc are best measured (Moonen and van Zanten 2021). A PhA greater than 6 is assumed normal in healthy people, although PhA was found to be significantly lower in women than in men and lower in the elderly (due to a decrease in muscle mass, which reduces Xc, and a decrease in water, which increases R) and infants (Barbosa-Silva et al. 2005; Mattiello et al. 2020). Proposed reference intervals for PhA at 50 kHz in healthy adult people are 6.87 ± 0.84 (5.57, 8.36 CI)° for women and 8.01 ± 0.85 (6.64, 9.48 CI)° for men. Values are expressed as mean ± SD and 5th and 95th percentiles (Barbosa-Silva et al. 2005). In this study, the mean ± SD PhA in our group of healthy, medium-sized breed donor dogs recorded at 50 kHz was 5.6 ± 1.2°. Further and larger studies in canine subjects could better establish PhA normality cut-off points and address whether they are similar among dog breeds and human cut-offs.

Since BIA methods have risen in popularity, there has been a documented unwillingness in human medicine to accept the limitations of the impedance method and the desire for performance beyond these limits. It must be noted that BIA itself is an indirect technique for body composition and body fluid volume assessment that could be affected by several factors related to study subjects and BIA methodology as well, such as age, sex, body fatness, electrode placement, room temperature, and physical activities to name a few (Kushner et al. 1996; Ward 2019). In this study, we standardized the protocol and tried to control as many variables and interferences as possible.

First, this study has been designed as an experimental model of acute blood loss. It was conducted on healthy, morphometrically homogeneous, fasted, medium-sized adult donor dogs under controlled conditions, with the donation timing and amount of blood drawn strictly monitored throughout the study period. In clinical practice, measurements of morphometric parameters, including weight, height, or body dimensions, are mainly used to reflect the amount of body fat and its distribution in the population under study. The classic BIA method includes these morphometric parameters in predictive equations (validated against reference methods) to estimate body composition and body fluid volumes. Since morphometric measurements have been proven to be reproducible and accurate in dogs (Sutter et al. 2008), in our study, we adapted the BIA anthropometric measurements to dogs, according to previous studies (Yaguiyan-Colliard et al. 2015a, b) to better assess the homogeneity in morphometry, and consequently, the conductive properties of our study groups. Both groups were composed of medium-sized hunting dog breeds, and differences in morphometry between the two groups were not statistically significant (Table 1).

To overcome the lack of canine predictive equations and the limitations of the classic BIA approach in dogs, we conducted a longitudinal assessment of raw BIA parameters of Z, R, Xc, and PhA in our study groups. Therefore, we only detected and compared changes and trends of raw BIA parameters, without an exact estimation of body fluid volume variations.

However, this serial assessment of each study dog over a short period allowed us to overcome any possible intra and interindividual effects on the analysis. The intervals between the two BIA measurement sessions (before and after blood donation in donors and T0 and T1 in controls) were indeed the same for all the subjects in both groups and closely spaced (20 min), thus minimizing the effect on BIA data of any body composition changes that could have occurred if the two time point measurements had been more distant in time.

The mean significant increase of the PhA and all the other bioelectrical parameters in this experimental model of acute fluid imbalances suggests that serial BIA could be a useful monitoring tool in clinical practice. Although the present study describes an experimental model of acute hemorrhage rather than naturally occurring disease, bioelectrical results indicate that this method might be helpful in efficiently and safely monitoring spontaneous disease and tracking body fluid imbalances in other clinical conditions that could lead to water retention or loss.

## Conclusions

BIA was feasible, non-invasive, quick, safe, and well-tolerated by all study dogs. Measurements were carried out without sedation or anesthesia, and electrode crocodile clips used in this study were similar to those of an ECG. They didn’t create any discomfort for the animals. Real-time results, portability, and low cost emphasize a possible point-of-care application of the technique in veterinary medicine.

In this experimental model of acute hemorrhage, we investigated an acute loss of TBW from the intravascular space: the reduction of BIA raw parameters after a mean, acute, whole-blood loss of 299 ± 59 ml suggests that BIA could early detect a mean acute total blood volume loss < 20% in non-sedated dogs. Even if this experiment was performed on healthy blood donor dogs under controlled conditions, our results, along with the safeness of the method, hint that the evaluation of BIA parameters could be potentially helpful in monitoring spontaneous disease and monitoring body fluid imbalances also in other clinical conditions that could lead to water retention or loss. The serial, longitudinal assessment of BIA raw parameters could be potentially suitable in dogs as it is in people to detect and monitor congestive states in clinical settings and provide, at the same time, helpful information about tailoring ongoing therapy. Further research on canine patients could also demonstrate and validate the biological meaning and the potential clinical application of the PhA as a biomarker of body fluid imbalances. Its longitudinal assessment could overcome the current lack of predictive equations for dogs, and it may be for dogs as it is a more reliable parameter for humans, rather than classic equation-based BIA, in situations of rapidly changing body fluid volumes. Future studies could establish and validate canine PhA cut-off points in health and disease.

## Supplementary Information

Below is the link to the electronic supplementary material.

Supplementary file1 (DOCX 323 kb)

## Data Availability

All relevant data are included in the manuscript.

## Abbreviations

BIA: Bioelectrical impedance analysis
CHF: Congestive heart failure
HCT: Hematocrit
RBC: Red blood cells
TBW: Total body water
Z: Impedance
R: Resistance
Xc: Impedance
PhA: Phase angle
BL: Body length
RC: Rib cage circumference
AC: Abdominal circumference
