# Supplementary figures and images for: Bioelectrical Impedance Analysis (BIA) detects body resistance increase in dogs undergoing blood donation
Source: Vet Res Commun. 2024 Sep 27;48(6):3889–97. doi: 10.1007/s11259-024-10555-1 (PMC11538226; doi:10.1007/s11259-024-10555-1)

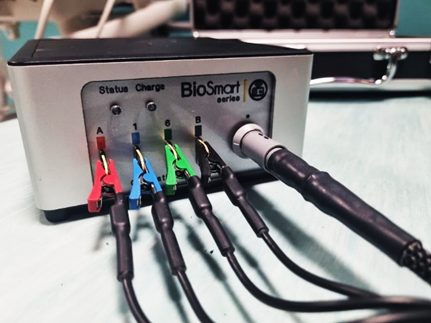


Figure 2. Biosmart ® EX.516(XX) calibration test.

Supplement: Supplementary file 1 — Supplementary file1 (DOCX 323 kb) [file 11259_2024_10555_MOESM1_ESM.docx]
